# CubicPat: Investigations on the Mental Performance and Stress Detection Using EEG Signals

**DOI:** 10.3390/diagnostics15030363

**Published:** 2025-02-04

**Authors:** Ugur Ince, Yunus Talu, Aleyna Duz, Suat Tas, Dahiru Tanko, Irem Tasci, Sengul Dogan, Abdul Hafeez Baig, Emrah Aydemir, Turker Tuncer

**Affiliations:** 1 Department of Digital Forensics Engineering, Technology Faculty, Firat University, Elazig 23119, Turkey; 211144203@firat.edu.tr (U.I.); 210509039@firat.edu.tr (Y.T.); 231144111@firat.edu.tr (A.D.); 231144106@firat.edu.tr (S.T.); 212144203@firat.edu.tr (D.T.); turkertuncer@firat.edu.tr (T.T.); 2 Department of Neurology, School of Medicine, Firat University, Elazig 23119, Turkey; itasci@firat.edu.tr; 3 School of Management and Enterprise, University of Southern Queensland, Toowoomba, QLD 4350, Australia; abdul.hafeez-baig@unisq.edu.au; 4 Department of Management Information Systems, Management Faculty, Sakarya University, Sakarya 54050, Turkey; emrahaydemir@sakarya.edu.tr

**Keywords:** cubic pattern, Directed Lobish, EEG mental performance detection, EEG stress detection, cortical connectome diagram, explainable feature engineering

## Abstract

**Background\Objectives:** Solving the secrets of the brain is a significant challenge for researchers. This work aims to contribute to this area by presenting a new explainable feature engineering (XFE) architecture designed to obtain explainable results related to stress and mental performance using electroencephalography (EEG) signals. **Materials and Methods:** Two EEG datasets were collected to detect mental performance and stress. To achieve classification and explainable results, a new XFE model was developed, incorporating a novel feature extraction function called Cubic Pattern (CubicPat), which generates a three-dimensional feature vector by coding channels. Classification results were obtained using the cumulative weighted iterative neighborhood component analysis (CWINCA) feature selector and the t-algorithm-based k-nearest neighbors (tkNN) classifier. Additionally, explainable results were generated using the CWINCA selector and Directed Lobish (DLob). **Results:** The CubicPat-based model demonstrated both classification and interpretability. Using 10-fold cross-validation (CV) and leave-one-subject-out (LOSO) CV, the introduced CubicPat-driven model achieved over 95% and 75% classification accuracies, respectively, for both datasets. **Conclusions:** The interpretable results were obtained by deploying DLob and statistical analysis.

## 1. Introduction

The cortical connectome diagram (CCD) aims to represent the functional and structural connections of the brain, and it is designed for use in neuroscience [1]. Mapping the interactions between different brain regions provides valuable insights into the neural basis of various cognitive and emotional processes [2,3]. This approach allows researchers to uncover the complex dynamics of brain activity and their effects on mental health and performance [4,5].

This study constructed CCD using electroencephalography (EEG) signals and a new symbolic language known as Directed Lobish (DLob) [6]. This method enables the systematic encoding of EEG data into symbolic sequences, facilitating the identification of patterns associated with different neural states [7]. The generated connectome diagrams visualize these patterns and quantify their complexity through metrics, such as information entropy, providing an explanatory framework for understanding brain function [8].

Integrating the cortical connectome with DLob increases its usefulness in distinguishing between mental performance and specific cognitive and emotional states, such as stress. For example, high mental performance was characterized by dynamic interactions across multiple brain lobes, particularly in the frontal region, as highlighted by entropy values [9]. Conversely, stress detection exhibited more predictable patterns with lower entropy, highlighting distinct neural signatures associated with these states [10]. Using this innovative methodology, the study bridges the gap between raw EEG data and actionable neuroscientific insights. CCD serves as a powerful visualization tool that offers not only high classification accuracy but also the interpretability needed to advance the fields of cognitive neuroscience and mental health diagnostics [11].

This study utilized EEG datasets to examine two distinct conditions: mental performance and stress detection. Mental performance datasets involved tasks resembling IQ tests, highlighting neural patterns associated with high cognitive functioning. Conversely, the stress detection dataset captured neural responses to earthquake-related stressors. By leveraging a new feature engineering framework, termed Cubic Pattern (CubicPat), the study achieved high classification accuracies and provided interpretable results, bridging the gap between computational models and neuroscientific insights.

### 1.1. Literature Review

Many different machine learning techniques have been presented in the literature [12,13,14]. Some of the studies conducted on stress and mental performance detection in the literature are given below.

Cambay et al. [7] presented the QuadTPat-based model for stress detection using EEG signals from 310 earthquake-affected participants. Using innovative feature extraction and classification methods, their model achieved 92.94% accuracy in 10-fold cross-validation. The study highlighted the critical role of the frontal and temporal lobes in stress responses and advanced explainable artificial intelligence in neuroscience. Mane et al. [15] developed an approach for mental stress detection using EEG signals. Their study employed EEG datasets from 28 individuals under controlled stress-inducing conditions, including auditory and visual stimuli. Their methodology, integrating wavelet transform for feature extraction and machine learning algorithms for classification, achieved an average accuracy of 93.2%, demonstrating the effectiveness of EEG-based imaging for stress detection. Marthinsen et al. [16] proposed a cost-effective approach for psychological stress detection using an optimized 8-channel EEG setup, reducing the standard 32-channel configuration. Data from 28 subjects, captured during real-life and arithmetic-induced stress scenarios, were analyzed using machine learning techniques. The study achieved 87.5% accuracy with wavelet scattering features and an SVM classifier. Patel et al. [17] presented a hybrid deep learning approach for mental stress detection using EEG signals from the DEAP dataset, which included recordings of 32 participants exposed to emotion-inducing videos. By combining one-dimensional CNN with a bidirectional long- and short- term memory network, their study achieved a high classification accuracy of 88.03%, outperforming conventional machine learning methods. Hafeez and Shakil [18] developed an EEG-based approach to classify mental stress levels using deep learning. EEG data from 14 students were collected during mental arithmetic tasks with and without time constraints to induce varying stress levels. The study achieved classification accuracies of 70.67% using LSTM and 90.64% with CNN on brainwave images. Saini et al. [19] proposed a one-dimensional CNN for mental task classification using artifact-free and artifact-contaminated EEG signals from publicly available and in-house databases. Their study utilized EEG datasets, including the Keirn and Aunon database, EEGMAT database, and a newly recorded single-channel EEG database, to classify both binary and multiclass mental tasks. Their approach achieved subject-independent classification accuracies of up to 100% for specific task pairs in the Keirn database and 99% and 98% for the EEGMAT and recorded databases. Zeng et al. [20] suggested a model to classify driver mental states, specifically alertness and fatigue, based on EEG data. They used a dataset comprising 28,176 epochs of EEG signals recorded from 10 subjects during a driving simulation experiment, with states labeled as TAV3 (alert) and DROWS (fatigue). Their results demonstrated that the model outperformed both traditional models like SVM and LSTM, achieving average intrasubject and intersubject classification accuracies of 92.68% and 84.38%. Wu et al. [21] presented an approach for mental fatigue assessment using arbitrary single-channel EEG data combined with morphological features and an LSTM-CNN architecture. Their study involved EEG recordings from 37 male participants under conditions of rested wakefulness and after 24 h of sleep deprivation, encompassing both eye-opened and eye-closed states. Their proposed methods demonstrated superior classification performance, with the LSTM-CNN achieving a true positive rate of 97.02% and a false positive rate of 3.50%. Ye et al. [22] proposed a bootstrap-aggregating ensemble CNN for identifying mental fatigue levels based on EEG features extracted from 14 channels during a language understanding task. Their study utilized EEG signals from 15 participants, processed to extract temporal statistics, power spectral density, and entropy indicators. Their approach achieved a participant-specific classification accuracy of 87.69% when using features from all domains, surpassing classical and deep classifiers. Lee et al. [23] developed a multi-feature block-based CNN for continuous EEG decoding to classify pilots’ mental states, including fatigue, workload, distraction, and normal state. Their study utilized EEG data collected from seven pilots with over 100 h of flight experience, recorded during simulated flight scenarios designed to elicit specific mental states. Their proposed model achieved an average classification accuracy of 75.00% in offline analysis and detection accuracies of 72.00% for fatigue, 72.00% for workload, and 61.00% for distraction in pseudo-online analysis. Dairi et al. [24] presented an unsupervised deep learning approach for classifying five mental tasks from EEG signals using a dataset from Graz University of Technology. Their method combined artifact removal, quadratic time-frequency distribution features, and a deep belief network-based isolation forest, achieving AUC values above 98.00%. Jiang et al. [25] proposed a random forest-CNN method to detect pilots’ low situation awareness levels using EEG data from 25 pilots under poor visibility. Their model achieved 84.8% accuracy, surpassing standalone RF 78.10% and CNN 81.60%.

As can be seen from the literature above, most of the research used deep learning models to attain high classification performance [26,27], and these studies did not employ explainable methods. For automatic EEG classification models, explainable results are very important for obtaining artificial intelligence-based insights into the brain [28,29,30]. To achieve this, we have presented a new explainable feature engineering (XFE) model. In this XFE model, we introduced a new feature extraction function called the Cubic Pattern. By deploying the Cubic Pattern and Directed Lobish (DLob), we developed an XFE model, which has been tested on the EEG mental performance detection and EEG stress detection datasets. In this context, the XFE model was evaluated on two EEG datasets to demonstrate the general classification ability of the proposed model. By presenting this XFE model, we have introduced a lightweight, highly accurate, generalizable, and explainable EEG signal classification model.

### 1.2. Literature Gaps

- Although this study uses two binary classification datasets to address this limitation, it does not fully establish the generalizability of the proposed method to multiclass or more complex datasets. Further validation on diverse datasets, including those with multiclass labels and varying complexities, is needed to conclusively demonstrate its robustness [31].
- Deep learning (DL) models are at the forefront of machine learning [32,33,34,35]. However, they often suffer from high computational complexity [36], which can be a bottleneck in practical applications [37]. Moreover, extracting explainable, feature-based results from large DL models remains challenging [38,39].
- While many EEG signal classification studies initially focused only on classification results [40], there is now a growing emphasis on uncovering biological mechanisms underlying these classifications [41,42].

### 1.3. Motivation and Our Model

The essential motivation of the presented model is to fill the identified gaps in the literature.

To address the first gap, two newly collected EEG datasets have been used in this research as a testbed: (i) EEG mental performance detection and (ii) EEG stress detection datasets.

To fill the second gap, a feature engineering model has been presented. The model has a simple structure, making the analysis of classification results straightforward. Additionally, the recommended feature engineering model has linear time complexity.

To address the third gap, the Directed Lobish (DLob) symbolic language was integrated into the presented model, enabling the creation of interpretable results.

To fill these three gaps, we present a new channel coding-based feature extraction function. A good machine learning model extracts features with high classification ability. To achieve this, the Cubic Pattern (CubicPat) is introduced. Each feature generated by CubicPat contains information from three channels. In this way, both classification and interpretable results are extracted using CubicPat, which is a critical method for creating the recommended explainable feature engineering (XFE) model.

The presented XFE model uses CubicPat to extract features. The most discriminative of the generated features have been selected by cumulative weighted iterative neighborhood component analysis (CWINCA) [43], and these features have been utilized as input for the t-algorithm-based k-nearest neighbors (tkNN) [11] classifier. In this research, both 10-fold and leave-one-subject-out (LOSO) cross-validation (CV) techniques have been used to validate the results of the tkNN classifier. To obtain explainable results, the indices of the chosen features selected by the CWINCA selector and the DLob symbolic language have been utilized. At this point, the recommended CubicPat-based XFE model generates both classification and explainable results.

### 1.4. Novelties and Contributions

Novelties:

- Two newly collected EEG signal datasets have been utilized as testbed.
- In this work, a new feature extraction function, which is CubicPat has been presented.
- To obtain both classification and explainable results from the CubicPat, a new CubicPat-based XFE model has been presented by integrating the CWINCA selector, tkNN classifier and DLob symbolic language.

Contributions:

- This research presents a lightweight feature engineering model for EEG signal classification. The proposed CubicPat feature extractor has been developed to generate informative features. The most discriminative of these features has been chosen using the CWINCA selector and these features have been classified with the tkNN classifier. To showcase robustness and reliability, both 10-fold cross-validation (CV) and leave-one-subject-out (LOSO) CV have been employed. The introduced CubicPat-related XFE model yielded over 95% classification accuracy with 10-fold CV and over 75% with LOSO CV for both EEG datasets. These results and findings openly demonstrated that the introduced CubicPat-driven XFE model contributes to feature engineering.
- The integration of the DLob symbolic language enables the generation of explainable results from the selected features. The DLob strings provide findings into cortical lobe activity. Statistical analysis of the DLob strings facilitated the creation of cortical connectome diagrams, and the created cortical connectome diagrams provide visual neural interaction. This contributes to the understanding of the biological mechanisms of the tested situations. Therefore, this research contributes to neuroscience since we have presented feature engineering-based findings in this research.

## 2. Materials and Methods

### 2.1. Material

To create a testbed for this research, two new EEG signal datasets were used, and both EEG datasets were collected using two different brain caps. The EEG stress dataset was collected with a 14-channel brain cap, and the EEG mental performance dataset was collected with a 32-channel brain cap. The details of these datasets are given below.

#### 2.1.1. EEG Mental Performance Detection Dataset

In this dataset, the researchers used a mental test with 60 questions, which is similar to an IQ test. The main objective of this dataset is to detect good and poor mental performance using EEG signals. Therefore, the EEG signals of the participants were collected while they were solving the mental capacity test. In this test, each question carries equal weight, with each question worth 2 points. Thus, the maximum possible score for the test is 120 points. For labeling, a threshold score of 90 was used. EEG signals corresponding to scores below 90 were labeled as low, while those with scores of 90 or higher were labeled as high.

The researchers used the Emotiv Flex 2 Saline—32 Channel EEG Head Cap System. The brain cap followed the 10/20 EEG placement system, and the sampling frequency of the collected EEG signals was 256 Hz. The channels used in this brain cap are: (1) Cz, (2) Fz, (3) Fp1, (4) F7, (5) F3, (6) FC1, (7) C3, (8) FC5, (9) FT9, (10) T7, (11) CP5, (12) CP1, (13) P3, (14) P7, (15) PO9, (16) O1, (17) Pz, (18) Oz, (19) O2, (20) PO10, (21) P8, (22) P4, (23) CP2, (24) CP6, (25) T8, (26) FT10, (27) FC6, (28) C4, (29) FC2, (30) F4, (31) F8, and (32) Fp2.

In this dataset, there are 3949 EEG samples (low: 2748 and high: 1201), and the length of each EEG sample is 15 s. Moreover, this dataset was collected from 55 participants, of whom 37 (32 males, 5 females) were labeled as low and 18 (15 males, 3 females) were labeled as high in mental performance.

#### 2.1.2. EEG Stress Detection Dataset

In this dataset, the primary objective is to detect stress caused by the earthquake, and the participants in this dataset were affected by the Great Turkey Earthquake Series on 6 February 2023. To induce earthquake-related stress, the researchers showed real earthquake videos to 150 participants. Additionally, meditation videos were shown to 160 participants. In total, EEG signals were collected from 310 participants, of whom 42 were women and the remaining 268 were men. While watching the videos, EEG signals were collected using the Emotiv Epoch X brain cap. This brain cap has 14 channels, and these channels are: (1) AF3, (2) F7, (3) F3, (4) FC5, (5) T7, (6) P7, (7) O1, (8) O2, (9) P8, (10) T8, (11) FC6, (12) F4, (13) F8, and (14) AF4. The sampling frequency of this brain cap is 128 Hz, and the length of each EEG segment is 15 s. In the EEG stress detection dataset, 1757 EEG segments are labeled as stress, and 1882 are labeled as control. In total, there are 3667 EEG signals in this dataset.

### 2.2. The Proposed Explainable Feature Engineering Model

To automatically classify the EEG signal datasets, an innovative XFE model has been presented. The presented XFE model is simple and highly accurate. Moreover, explainable results have been obtained using our model. The main objective of the recommended XFE is to demonstrate the classification and explainable results generation capability of the presented CubicPat feature extractor. Therefore, we have used CWINCA to choose the most distinctive features, tkNN was utilized to obtain classification results, and DLob was utilized to create explainable results.

**Figure 1:**
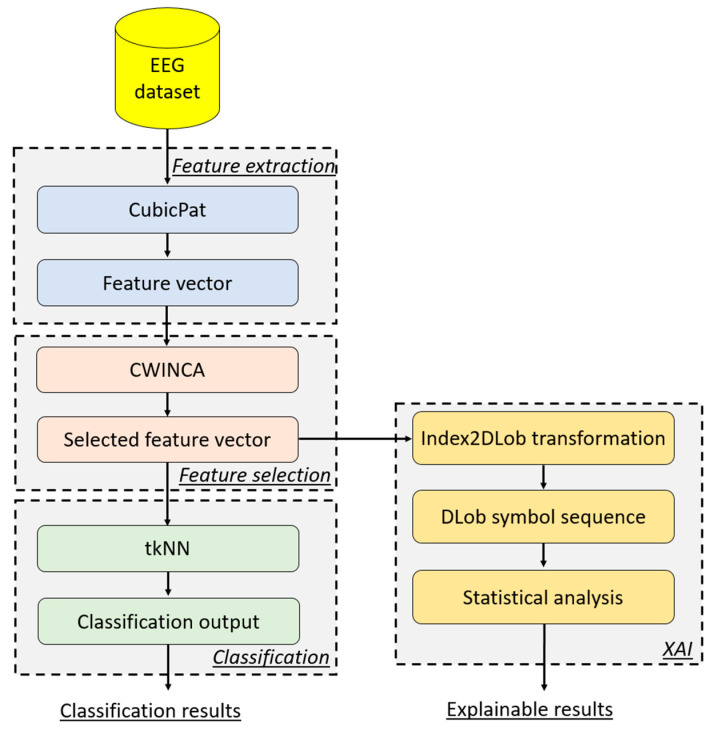
The schematic overview of the presented CubicPat-based XFE model.

The general block diagram of the recommended CubicPat-related XFE model is shown in Figure 1.

The details of the recommended CubicPat-based XFE model are provided below.

#### 2.2.1. The Recommended Feature Extraction Based on CubicPat

The main feature extraction function of the presented model is the recommended CubicPat feature extractor. This feature extractor is simple and effective, and the steps of the presented CubicPat are given below.

Step 1: Read the channels of each point.

(1) $$C^{i}=EEG\left(i,1:n\right),\;i\in \left\{1,2,\ldots ,ln\right\}$$

where C: channel vector, n: the number of the channels, and ln: the length of the EEG signals.

Step 2: Apply sorting in descending order.

(2) $$id=argsort\left(-C^{i}\right)$$

where id: the sorted indices of the channels.

Step 3: Apply cubic coding to generate feature vectors.

(3) $$map=\left(id\left(j\right)-1\right)\times n^{2}+\left(id\left(j+1\right)-1\right)\times n+\left(id\left(j+2\right)-1\right)$$

$$j\in \left\{1,2,\ldots ,L-2\right\}$$

Herein, map: the coded feature map signal value and L: length of the sorted indices of the channel.

Step 4: Compute the histogram value.

(4) $$histo\left(map+1\right)=histo\left(map+1\right)+1$$

Herein, histo: the histogram of the generated map value.

In this research, the extracted histogram has been utilized as a feature vector and the length of the feature vector is $n^{3}$. For 32 and 14 channeled EEG signals, the lengths of the feature vectors are 32,768 (=32^3^) and 2744 (=14^3^), respectively.

The pseudocode of the recommended CubicPat-based feature extractor is demonstrated in Algorithm 1.
**Algorithm 1.** The routine of the recommended CubicPat.**Input:** EEG signal with a length of ln. **Output:** Feature vector (histo) with a length of $n^{3}$. 01: **for** i = 1 to ln **do** 02:           **for** k = 1 to n **do** 03:                     $C^{i}\left(k\right)=EEG\left(i,k\right)$; 04:           **end for k** 05:           $id=argsort\left(-C^{i}\right)$
06:           **for** j = 1 to L−2 **do** 07:                     $map=\left(id\left(j\right)-1\right)\times n^{2}+\left(id\left(j+1\right)-1\right)\times n+\left(id\left(j+2\right)-1\right)$; 08:                     $histo\left(map+1\right)=histo\left(map+1\right)+1$; 09:           **end for j** 10: **end for i**

#### 2.2.2. Feature Selection

For feature selection, an iterative and effective feature selector is needed. Therefore, the CWINCA [43] feature selector has been utilized. The CWINCA feature selector is a developed version of the NCA [44] and INCA [45] feature selectors. By deploying cumulative weight computation, the range of the loop has been determined. In the iterative feature selection, the classification accuracy of each selected feature vector has been computed. Based on the computed classification accuracy, the best-selected feature vector has been chosen. The steps of the CWINCA feature selector used are given below.

Step 5: Compute the qualified identities of the generated features.

(5) $$w=\pi \left(f,y\right)$$

(6) $$idx=argsort\left(-w\right)$$

Herein, w: weight of the features (f), $\pi \left(.\right)$: NCA feature selection function, y: the real outcomes/labels, and idx: the qualified indices of the indices.

Step 6: Calculate the range of the loop by deploying cumulative weight. In this step, the start and stop values of the loop are computed. For this research, 0.75 and 0.99 threshold values are used. The 0.75 threshold was utilized to compute the start value of the loop, and 0.99 was used to determine the stop index.

(7) $$sv=CW\left(w,idx,0.75\right)$$

(8) $$fv=CW\left(w,idx,0.99\right)$$

Herein, sv: start value, fv: final value and $CW\left(.\right)$: cumulative weight computation function. In this step, loop range of the iteration is defined.

Step 7: Apply iterative feature selection.

(9) $$sf^{a-sv+1}\left(d,\;r\right)=f\left(d,idx\left(r\right)\right),\;a\in \left\{sv,sv+1,\ldots ,fv\right\},$$

$$r\in \left\{1,2,\ldots ,a\right\},d\in \left\{1,2,\ldots ,no\right\}\;\;(L2)$$

Here, sf: selected feature vector in the loop and no: number of observations.

Step 8: Compute the classification accuracy of each selected feature vector.

(10) $$co^{a-sv+1}=C\left(sf^{a-sv+1},y\right)$$

$$ca\left(a-sv+1\right)=\psi \left(co^{a-sv+1},y\right)\;\;(L2)$$

Herein, $C\left(.\right)$: the used classifier to calculate classification outcome (co) of each selected feature vector, ca: the classification accuracy and $\psi \left(.\right)$: the classification accuracy computation function.

Step 9: Choose the most accurate selected feature vector based on the computed classification accuracy. In this step, the greedy algorithm is used to select the best feature vector. In this regard, this function is a self-organized feature selector.

(11) $$bs=sf^{argmax\left(ca\right)}$$

where bs: the best of the selected feature vector.

#### 2.2.3. Classification

The tkNN classifier was proposed by Tuncer et al. [11] in 2024. This classifier uses loop-based parameter-changing, iterative majority voting (IMV) [46], and a greedy algorithm [47]. In this aspect, the tkNN classifier is an iterative and ensemble classifier. The steps of this classifier are given below.

Step 10: Compute parameter-based classification outcomes by changing parameters iteratively.

(12) $$pot_{s}=\kappa \left(bs,y,kv_{i},dist_{j},w_{k}\right),\;s\in \left\{1,2,\ldots ,30\right\}\;\;$$

$$\begin{array}{c} kv=\left\{1,2,\ldots ,5\right\},dist=\left\{CityBlock,Chebyshev,\;Euclidean\right\},\\ w=\left\{Inverse,\;Equal\right\}\;\; \end{array}$$

Herein, $\kappa \left(.\right)$: kNN classifier, pot: parameter-based outcome, kv: k value, dist: distance metric, and w: weight of the kNN. Tenfold CV and LORO CV were utilized as validations to generate these outcomes. In this step, 30 (=5 × 3 × 2) parameter-based outcomes have been generated by changing these parameters using nested loops.

Step 11: Deploy IMV to parameter-based outputs and generate 28 (=30 − 3 + 1) voted outcomes. The mathematical explanation of the IMV has been demonstrated below.

(13) $$ca\left(s\right)=\psi \left(pot_{s},y\right)$$

(14) $$dx=argsort\left(-acc\right)$$

(15) $$v_{q-2}=\varpi \left(parout_{dx\left(1\right)},parout_{dx\left(2\right)},\ldots ,parout_{dx\left(q\right)}\right),\;q\in \left\{3,4,\ldots ,30\right\}$$

where dx: the identities of the sorted outcomes and v: voted outcomes.

Step 12: Choose the final outcome among the generated 58 (=30 parameter-based + 28 voted) outcomes.

(16) $$ca\left(30+m\right)=\alpha \left(v_{m},y\right),m\in \left\{1,2,\ldots ,28\right\}\;\;$$

(17) $$indice=argmax\left(acc\right)$$

(18) $$fo=\left\{\begin{array}{c} pot_{indice},\;indice\leq 30\\ v_{indice-30},indice>30 \end{array}\right.$$

Here, indice: the identity of the maximum accuracy.

#### 2.2.4. Directed Lobish-Related Explainable Results Generation

The final phase of the presented model is the generation of DLob-related explainable results. Two different EEG signal datasets have been used in this research, collected using 32-channel and 14-channel EEG brain caps. Therefore, each channel has been coded using the DLob symbols. First, we have defined the DLob symbols below.

FL: Associated with logical thinking and analytical processes, key in planning and decision-making, as well as speech generation.

FR: Engaged in creative thought, spatial awareness, and emotional regulation, especially for interpreting nonverbal cues.

Fz: Involved in managing executive functions, focusing attention, and monitoring behavior, particularly during decision-making.

TL: Linked to language comprehension and auditory processing, playing a crucial role in memory related to verbal information.

TR: Processes nonverbal auditory input and contributes to memory, emotion, and recognition of complex auditory patterns like music.

PL: Integrates sensory information and handles tasks related to language processing, problem-solving, and logical reasoning.

PR: Plays a role in spatial awareness and the perception of patterns, crucial for navigating and processing the environment.

Pz: Important for sensory integration, body awareness, and coordination, contributing to overall attention and consciousness.

OL: Primarily involved in visual processing related to the right side of the visual field, including recognizing letters and words.

OR: Handles visual information from the left side of the visual field, aiding in spatial orientation and scene recognition.

Oz: Central to basic visual perception, processing input from both visual fields to integrate and interpret visual data.

CL: Manages motor control on the right side of the body and processes sensory input from that side, aiding fine motor skills.

CR: Controls left-side motor functions and processes sensory input from the left, coordinating body movements and touch sensations.

Cz: Integrates motor and sensory information from both sides of the body, crucial for coordinated movement and somatosensory processing.

Using the above DLob symbols, two look-up tables (LUTs) have been created to represent channels with these symbols. The LUTs are provided below:

LUT32: {Cz, Fz, FL, FL, FL, FL, CL, FL, FL, TL, CL, CL, PL, PL, PL, OL, Pz, Oz, OR, PR, PR, PR, CR, CR, TR, FR, FR, CR, FR, FR, FR, FR}.

LUT14: {FL, FL, FL, FL, TL, PL, OL, OR, PR, TR, FR, FR, FR, FR}.

Using these LUTs, we have created the DLob string by following the steps below.

Step 13: Generate DLob symbols from the indices of the selected features. Here, digit separation has been used to obtain the DLob symbols, as the indices of the LUTs must be generated to acquire the DLob symbols. Moreover, each feature of the recommended CubicPat includes three channel values. By utilizing the channel values and LUTs, the DLob symbols have been created.

(19) $$value=idx\left(w\right),\;w\in \left\{1,2,\ldots ,NoF\right\}$$

(20) $$ch_{j}=\frac{value-1}{NoC^{j-1}}\left(mod\;NoC\right)+1,\;j\in \left\{1,2,3\right\}$$

(21) $$Seq\left(c+j\right)=LUT\left(ch_{j}\right),\;c\in \left\{0,3,\ldots ,3\times NoSf-3\right\}$$

Here, value: value of the identity of the selected feature, NoC: number of channels, Seq: the DLob symbol sequence, LUT: look-up-table, and NoSf: the number of selected features.

Step 14: Obtain the statistical results of the generated DLob sequence by computing the transition of the symbols used, the histogram of the utilized symbols, and the information entropy of the generated DLob sequences.
Step 15: Train a large language model (LLM) using the information from the DLob (we have used a custom LLM) and our findings. Then, generate the explainable results for the generated DLob sequence. The custom LLM used is the Lobish EEG Interpreter (URL: https://chatgpt.com/g/g-E3Gvijurs-lobish-eeg-interpreter accessed on 1 October 2024).

These 15 steps define the presented CubicPat-based XFE model.

## 3. Experimental Results

The experimental setting and results are provided in this section. The presented CubicPat-based XFE model was introduced to obtain both classification and explainable results. Therefore, we used CubicPat, CWINCA, tkNN, DLob, a custom LLM (custom GPT 4o; https://chatgpt.com/ accessed on 1 October 2024), and statistical analysis. Except for the custom GPT, the entire model was programmed using the MATLAB 2024a programming environment, and a simply configured laptop was used to program the CubicPat-based model. In MATLAB 2024a, we created .m files for implementing CubicPat, CWINCA, tkNN, the DLob sequence generator, and statistical analysis. These functions were called using the main function, and the results were obtained. The presented model is a parametric XFE model, and the parameters used for this model are tabulated in Table 1.

Using the above parameters (see Table 1), both classification and interpretable/explainable results were obtained for EEG mental performance detection and EEG stress detection.

### 3.1. Classification Results

**Figure 2:**
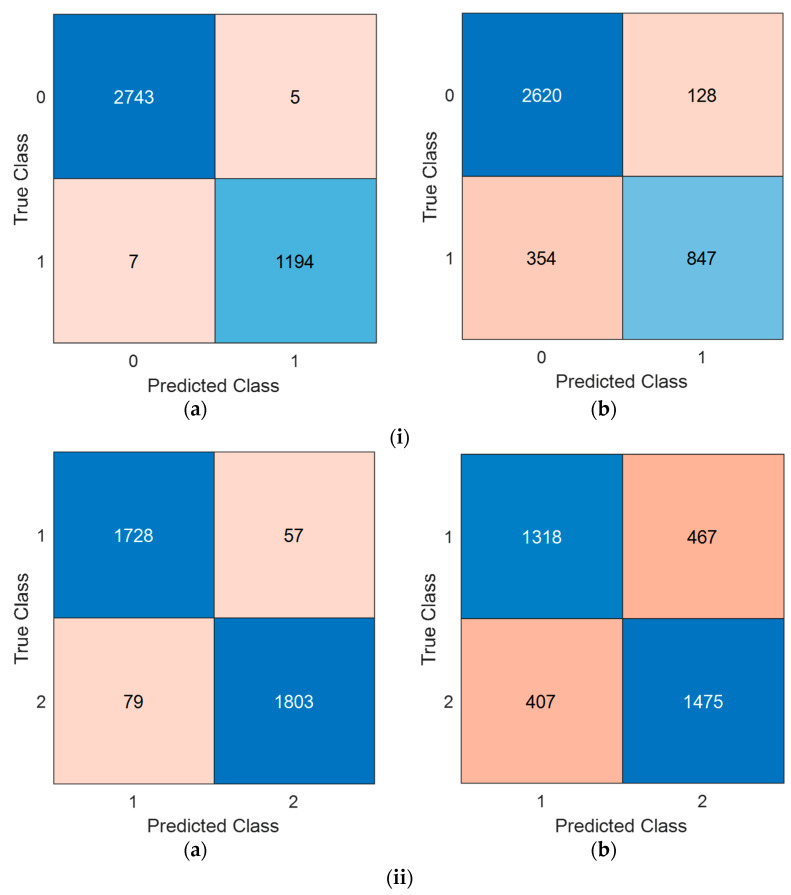
The confusion matrices of the recommended CubicPat-based XFE model. (**i**) EEG mental performance detection dataset. Herein, 0: Low performance, 1: High performance; (**a**) 10-fold CV; (**b**) LOSO CV (**ii**) EEG stress detection dataset. Herein, 1: Stress, 2: Control; (**a**) 10-fold CV; (**b**) LOSO CV.

In this work, we used two EEG signal datasets: (i) EEG mental performance detection and (ii) EEG stress detection. The classification results of the presented CubicPat-based XFE model were computed using the tkNN classifier, which employed two validation techniques: (1) 10-fold CV and (2) LOSO CV. In this aspect, four classification results were generated for both datasets. Moreover, classification accuracy, F1-score, and geometric mean metrics were utilized to measure classification performance. The confusion matrices were created to compute these performance metrics, and the matrices are showcased in Figure 2.

Based on the computed confusion matrices, the performance evaluation metrics have been tabulated in Table 2.

Table 2 showcases that the proposed CubicPat-based XFE model achieved classification accuracies of 99.70% and 96.29% with a 10-fold CV on the mental performance and stress datasets, respectively. Additionally, accuracies of 87.79% and 76.17% were obtained with LOSO CV on the mental performance and stress datasets, respectively. Furthermore, for a 10-fold CV, the recommended CubicPat-based XFE model achieved over 95% classification performance across all three performance evaluation metrics. Classification performances exceeding 75% were computed with LOSO CV across all three performance evaluation metrics.

### 3.2. Explainable Results

The second result of the presented CubicPat-based XFE model is the explainable results. To obtain these explainable results, we used a DLob-based XAI method, and the explainable results were extracted for both EEG signal datasets. These results are valuable for neuroscience.

To verify the order of the generated DLob symbols, it must first be confirmed that the feature indices used to obtain the DLob symbols are correctly mapped to their corresponding channels via the appropriate LUT (i.e., LUT32 or LUT14, since two datasets were used in this research, collected using 14- and 32-channel brain caps). The identities of the selected features and LUTs have been used to extract the DLob symbols. This involves checking that the step separation correctly identifies the three channels from each feature value and that these channels generate the correct DLob symbols in the same order when referenced with respect to the LUT, because the features generated with the principal CubicPat contain three-channel information. Each of the resulting DLob symbols represents a cortical lobe. To quantify the activation of these cortical lobes in the specified states, a histogram of the resulting DLob string is extracted, and then the transition table of the generated DLob symbols is computed to create the cortical connectome diagram. Moreover, the information entropy of the generated DLob symbols is computed using the generated DLob symbol histogram. In addition to these steps, the substrings/patterns obtained from the DLob sequence are interpreted with the help of the custom LLM created. For example, a pattern containing FRTROR DLob symbols indicates that cognitive, sensory, and visual activities occur on the right side of the brain.

In the first steps, DLob sequences were extracted. The generated explainable results are demonstrated below.

**Figure 3:**
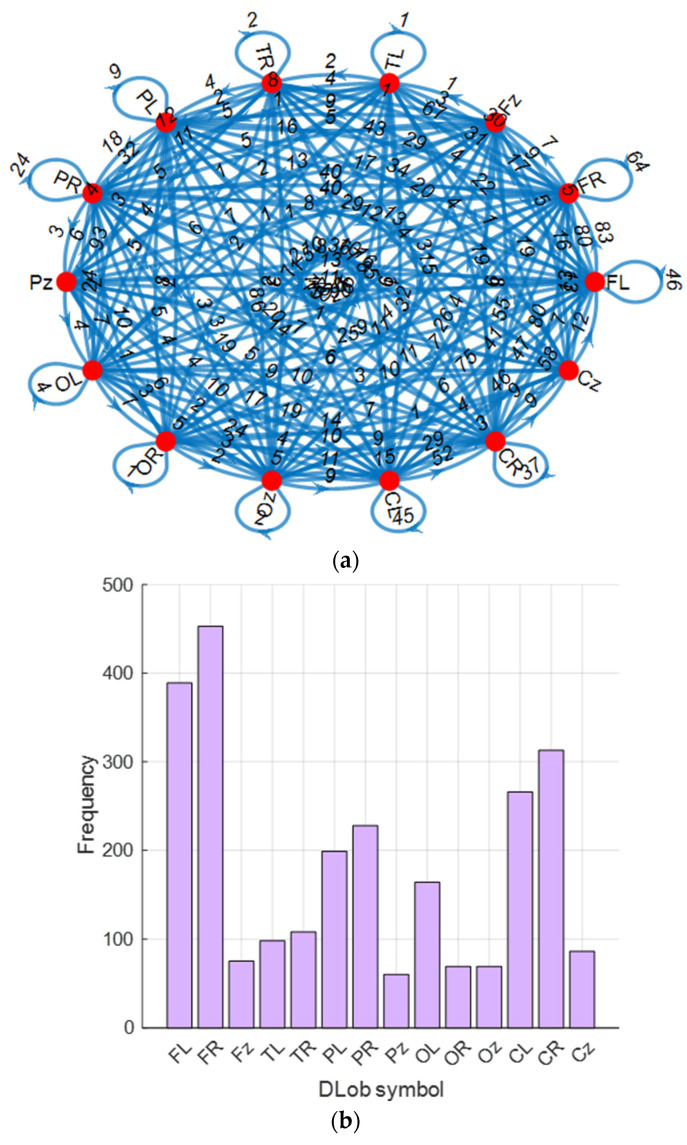
The generated cortical connectome diagram and histogram of the symbols of the mental performance detection; (**a**) Cortical connectome diagram; (**b**) Histogram of the symbols.

For the EEG mental performance detection dataset, the generated connectome diagram and the extracted histogram are shown in Figure 3.

**Figure 4:**
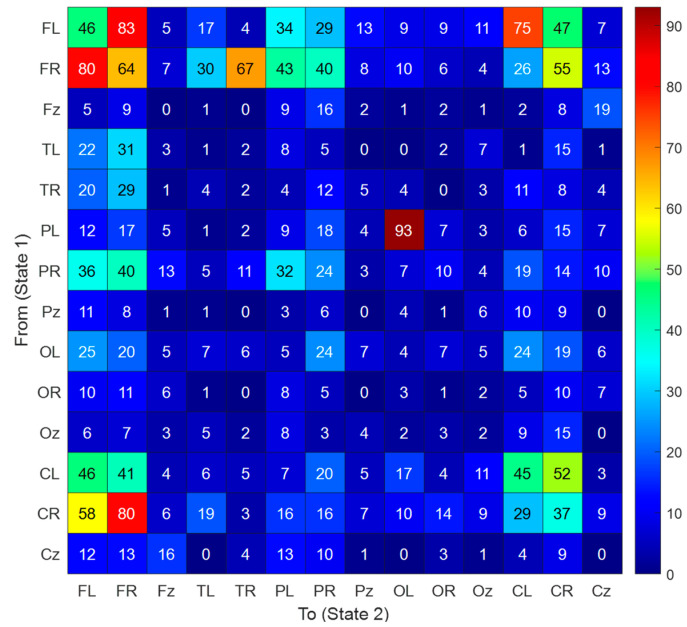
Transition table generated for mental performance detection.

Additionally, the computed transition table of the generated DLob symbols is showcased in Figure 4. Using this transition table, the displayed cortical connectome diagram was created.

In Figure 4, the highest transition computed is 93 between PL and OL. Additionally, the information entropy of the generated DLob sequence for mental performance detection computed is 3.4974.

In the stress detection dataset, a 14-channel brain cap was utilized, and to represent these channels, eight DLob symbols—FL, FR, TL, TR, PL, PR, OL, and OR—were used. The computed explainable results are demonstrated below.

**Figure 5:**
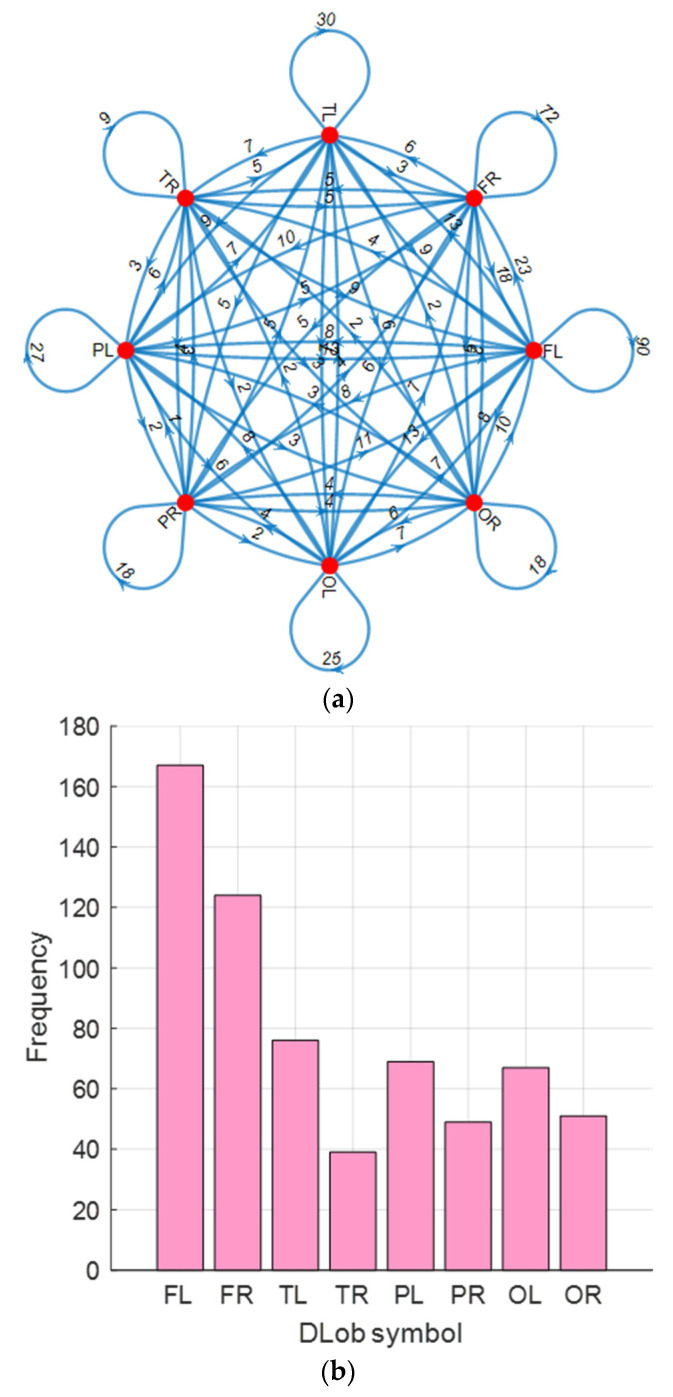
Cortical connectome diagram and DLob symbols’ histogram of the stress detection. (**a**) Cortical connectome diagram (**b**) Histogram of the symbols.

In Figure 5, the explainable results for the generated DLob symbols for stress detection are demonstrated. The cortical connectome diagram was created using the transitions of the symbols, and the histogram of the DLob symbols was used to compute the information entropy of the obtained DLob sequence for stress detection. The information entropy of the generated DLob string is 2.8331. First, the transitions of the DLob symbols for stress detection have been tabulated in Table 3.

According to Table 3, the highest transition is between FR and FR, and it was computed as 57. This transition is highlighted in bold font. Moreover, there is no TR symbol. Additionally, the information entropy of the created DLob sequence for stress detection was calculated as 2.8331.

## 4. Discussion

**Figure 6:**
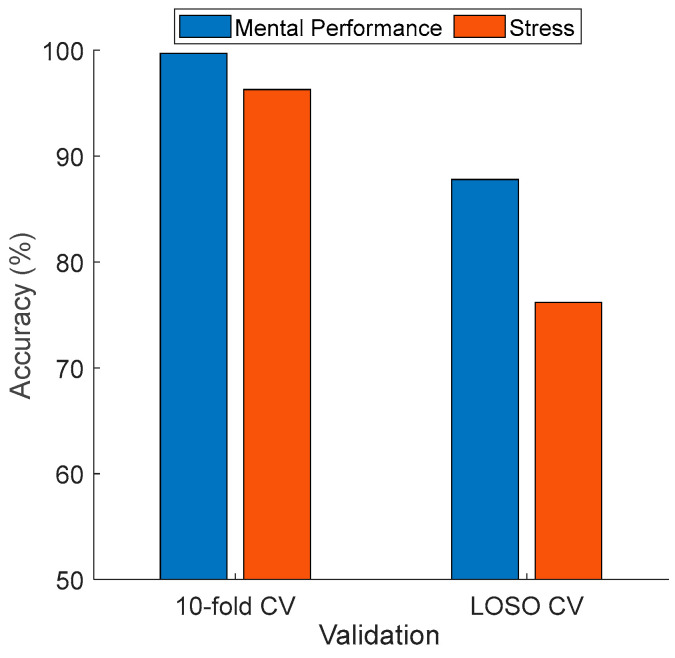
Comparison of the two used datasets.

The recommended CubicPat-based XFE model generates both classification and explainable results. As stated in Section 4, the recommended model attained over 75% classification performance for both datasets. In the first dataset, the EEG signals were collected using a 32-channel brain cap. For the other dataset (stress detection), EEG signals were collected using a 14-channel brain cap. To compare the classification performance of the introduced CubicPat-based XFE model, the accuracies of these datasets were compared, and this comparison is showcased in Figure 6.

According to Figure 6 and Table 2, our presented CubicPat-based XFE model yielded higher classification performance on the EEG mental performance dataset than on the stress detection dataset. The reasons for these results are as follows: To collect EEG signals for the mental performance detection dataset, a 32-channel brain cap was used, while a 14-channel brain cap was used to collect the stress detection data. For the stress detection dataset, earthquake videos were shown to participants, and the researchers asked, “Are you stressed?” Based on the participants’ responses, the EEG stress detection dataset was labeled. In contrast, for the EEG mental performance detection dataset, the researchers administered a mental test to participants, collected EEG signals while they solved the test, and labeled the EEG observations based on the participants’ scores. Therefore, our model achieved high classification performance on the mental performance detection dataset.

The recommended model reached satisfactory classification performance on both datasets because the presented CubicPat generates features by capturing the relationships between channels, and two self-organized methods (CWINCA and tkNN) were integrated to obtain classification results.

To demonstrate the high classification ability of the recommended CubicPat-based XFE model, a comparative results table is presented. In this table (Table 4), the results of our model are compared to those of state-of-the-art (SOTA) models.

According to the results of Table 4, the recommended model attained satisfactory classification performance. Therefore, the recommended model is valuable for EEG signal classification. Moreover, four different results have been computed. This shows that the presented model has general EEG signal classification capability.

The presented CubicPat-based model also generates interpretable results. The obtained interpretable results have been discussed below for both datasets.

### 4.1. Interpretable Results Discussions of the Mentalperformance Dataset

For this dataset, the information entropy of the generated DLob string is 3.4974, and this information entropy is close to the maximum entropy, which is 3.8074 (=log_2_14). Moreover, the generated DLob string contains all the defined DLob symbols. This entropy value demonstrates the complexity of the mental performance. Additionally, the dominance of the frontal lobe highlights the importance of the frontal lobe’s role in mental performance detection. Using generative AI, our generated DLob symbols have been discussed below with respect to mental performance detection. Some patterns have been discussed below to explain mental performance detection clearly. We have showcased some DLob arrays derived from the mental performance DLob string.

CzFLFRCzOzCLCzPRPLCzCRCRCz: This pattern showcases the brain is managing attention, logical tasks, and sensory input simultaneously.

TRFRCzFRPLCzFRPLCzFLTLCzFRFLCzFRCz: This combination indicates the brain is switching between emotional and logical processes during cognitive challenges. Moreover, there are transitions from right to left and central areas.

CLCRCzFLPLCzFLCRCzOzCRCzCRFRCzFLPRCz: This pattern defines CL and Cz, showing continuous attentional focus and hemispheric transitions. The frequent activation of Oz and CR indicates visual processing coupled with motor planning or decision-making. The involvement of FL and PR showcases decision-making based on sensory inputs.

TLTRCzTLFRCzCLCRCzOLFRCzCzFRCzFLPRCz: The TL and TR define auditory processing and memory retrieval. Cz and FL again showcase logical decision-making. The OL activation highlights visual processing. This pattern is indicative of a brain adapting to tasks requiring both sensory integration and executive control.

FRPRCzCRFLCzCLFRCzTLFLCzTRFLCz: This sequence showcases cognitive flexibility and adaptability under mentally demanding conditions.

### 4.2. Interpretable Results Discussions of the Stress Dataset

By using this dataset, the information entropy of the generated DLob string was computed as 2.8331, and eight DLob symbols (the maximum entropy value is 3 = log_2_8) were used. Therefore, stress detection is more predictable than mental performance detection. Moreover, the generated DLob string did not contain the TR symbol. To provide more explainable results, some patterns/sequences of the generated DLob string have been discussed below.

TLTROLPLTRPRTLPL: This pattern begins with TL and TL represents emotional processing, language-based activities, and memory retrieval. These activations are valuable for an earthquake victim. TL transitions to TR and this showcases the brain’s immediate response to environmental sounds during an earthquake. By using PL and PR, sensory inputs such as body coordination are activated.

FLFLFLTRFLFLPRTRFRFRFRFROLORFLFLTLPLFLFL: The alternation between FL, FR, and OR reflects a heightened state of cognitive, emotional, and visual processing.

FRFLFRFRFLPLFRTLTLOLPLOLFRFRTLOLFRFRPLOROLTL: This pattern indicates dynamic transitions between logical reasoning, sensory integration, and emotional responses.

FRFROLORFLORPRFLFLFLOROLPLOLORPRTLTRTLTRTL: This sequence reflects the brain’s constant switching between emotional regulation, visual scanning, and auditory processing, characteristic of an earthquake scenario.

### 4.3. Causal Connectome Theory

We have presented a lobe transition table to explain our new causal connectome theory (CCT) in Table 5.

By using this table, CCT has been explained using the obtained transitions.

### 4.4. Highlights

The findings, advantages, limitations, and future works have been explained below.

Findings:

- The CubicPat-based XFE model achieved over 95% classification accuracy using 10-fold CV and over 75% accuracy using LOSO CV for both EEG mental performance detection and stress detection datasets.
- For EEG mental performance detection: 99.70% accuracy with 10-fold CV and 87.79% accuracy with LOSO CV.
- For EEG stress detection: 96.29% accuracy with 10-fold CV and 76.17% accuracy with LOSO CV.
- CubicPat feature extraction generates a three-dimensional feature vector by coding EEG channels.
- For both datasets, cortical connectome diagrams were created using DLob symbol transitions.
- For mental performance detection, the DLob string entropy was 3.4974 (close to the maximum entropy of 3.8074). By using this entropy value, the complexity rate of the generated DLob sentence is computed as 91.86% (=3.4974/3.8074) and it is indicated as a complex and dynamic brain interaction. Moreover, there is a dominant FL activation in mental performance detection.
- For stress detection, the entropy was computed as 2.8331. For mental performance detection, eight DLob symbols were used. Therefore, the maximum entropy for it is computed as 3. Therefore, the complexity ratio of the generated DLob sentence for stress detection is 94.44% (=2.8331/3).
- For both cases (stress and mental performance detection), the most activated lobe is the frontal lobe.
- According to our findings, stress detection is a more complex process than mental performance detection.
- The frequency of PL-OL activation indicates that the brain focuses on environmental awareness, perception, and logical reasoning, all essential components of mental performance. Frequent PL-OL transitions reflect the complementary roles of sensory and visual information processing during mental performance. These activations enable the effective integration and interpretation of external stimuli, indicating that the brain is engaging in higher-level cognitive functions, such as reasoning, decision-making, and problem-solving. PL-OL activation was high in mental performance detection because EEG signals were collected during test-solving.
- For stress detection, the most frequent transition is FL-FL. It clearly depicts that stress is an internal process.
- The frontal lobe (FL and FR) plays a critical role in stress-related processes due to its involvement in decision-making, cognitive flexibility, and emotional regulation. Increased transitions from FL to itself (90) and from FL to FR (23) indicate heightened cognitive processing and emotional control attempts during stress, clearly highlighting that stress is an internal process.
- The temporal lobe (TL and TR), responsible for auditory processing and memory recall, shows moderate activation with FL and FR. This suggests that stress may trigger responses to auditory stimuli (e.g., earthquake sounds in this context). In addition, high TL activation may indicate that stress also affects emotional regulation.
- The parietal lobe (PL and PR), associated with spatial awareness and sensory integration, shows fewer transitions overall. Transitions in this lobe can be interpreted as being related to spatial awareness.
- The occipital lobe (OL and OR) shows particularly pronounced transitions from OL to OL (25). This likely reflects the role of visual processing during stress, influenced by the visual stimuli used in the experiment (earthquake videos).
- High self-transitions (e.g., FL to FL, FR to FR) may represent repetitive neural activity patterns characteristic of stress responses, involving cognitive overload or rumination.
- The higher entropy in DLob sequences for stress perception compared to mental performance perception reflects the complexity and high dynamism of neural interactions under stress. This finding aligns with the pathophysiological understanding that stress is a state of hypervigilance in neural networks.
- The DLob transition table captures these dynamics indirectly through higher FL and FR activity. Interhemispheric imbalances, such as differences in transitions between OL and OR (OL: 25, OR: 18), may reflect asymmetries in visual processing under stress.
- The EEG graphoelements showcase that this EEG corresponds to a person under stress. High self-transitions in the frontal lobe (FL to FL: 90, FR to FR: 72) reflect repetitive neural activity, which is typical of cognitive overload and rumination. Increased transitions between FL and FR (FL to FR: 23) show heightened communication for cognitive and emotional regulation. The occipital lobe (OL to OL: 25) exhibits strong self-transitions. Moderate temporal lobe activity (TL to TL: 30) suggests responses to auditory stimuli and emotional memory recall. Interhemispheric asymmetry in the occipital lobe (OL: 25 vs. OR: 18) indicates imbalanced visual processing. Finally, lower parietal lobe activation (PL to PL: 27, PR to PR: 18) suggests reduced spatial reasoning, as stress redirects cognitive resources to emotional and vigilance processes. In summary, high frontal and moderate temporal transitions are key indicators of stress, as shown by our findings.
- High transitions between FL and FR (FL to FR: 83, FR to FL: 80) indicate strong communication between hemispheres for cognitive functioning for mental performance detection.
- Self-transitions in the frontal lobe (FL to FL: 46, FR to FR: 64) reflect repetitive neural activity needed for focus and problem-solving for mental performance detection.
- There is a low (PL to PL: 9, PR to PR: 18) self-transition in the parietal lobe for mental performance detection.
- Moderate occipital lobe transitions (OL to OL: 24, OR to OR: 24) suggest occasional visual processing.
- Central lobe transitions (CL to CR: 52, CR to CL: 58) show bilateral coordination.
- The highest transition occurs between PL and OL (93), which depicts the strong sensory and cognitive interaction during mental performance tasks.
- The EEG graphoelements define that this EEG corresponds to a participant engaged in mental performance tasks. High transitions between the frontal lobes (FL to FR: 83, FR to FL: 80) highlight strong communication between hemispheres, which is essential for cognitive functioning. Self-transitions in the frontal lobe (FL to FL: 46, FR to FR: 64) reflect repetitive neural activity, necessary for focus and problem-solving. The parietal lobe shows lower self-transitions (PL to PL: 9, PR to PR: 18), suggesting minimal engagement in sensory integration. Moderate transitions in the occipital lobe (OL to OL: 24, OR to OR: 24) indicate occasional visual processing. Central lobe transitions (CL to CR: 52, CR to CL: 58) reveal bilateral coordination. In summary, high frontal activity and strong sensory-cognitive interactions are key indicators of mental performance, as shown by the findings.
- The proposed CubicPat feature extractor is presented to analyze EEG signals by extracting channel relationships. It employs a ranking-based transformer to identify changes corresponding to standard graphoelements observed in clinical neurophysiology, such as sharp waves, slow waves, and rhythmic oscillatory activity within standard EEG frequency bands (e.g., alpha [8–12 Hz], beta [13–30 Hz], theta [4–7 Hz], and delta [0.5–4 Hz]). Sharp waves may represent transient cortical activation, often associated with epileptic form activity, while theta rhythms are typically linked to cognitive tasks or drowsiness, depending on their regional prominence.
- By integrating the DLob symbolic language, the extracted features are mapped to specific cortical regions, enabling interpretations relevant to neurophysiology and clinical practice. For example, frontal lobe activations (FL and FR) are associated with decision-making and executive functions. High activity in the frontal lobe is often linked to cognitive engagement, such as problem-solving or working memory tasks. Temporal lobe activations (TL and TR), which are involved in auditory processing and memory encoding, indicate heightened activity during auditory tasks or emotional recall. Occipital lobe activations (OL and OR), responsible for visual processing, reflect increased attention during visually demanding tasks.
- Clinically, this approach helps physicians interpret EEG results in various conditions. For instance, sharp waves and spikes detected in the temporal lobe (e.g., TL and TR) may indicate focal temporal lobe epilepsy. Increased beta activity in the frontal lobe (FL and FR) can suggest heightened arousal or stress, as seen in anxiety disorders, while decreased alpha rhythms in the occipital region (OL and OR) may point to early neurodegenerative changes, such as those observed in Alzheimer’s disease.
  By combining the CubicPat feature extraction method with the DLob symbolic framework, the proposed XFE model not only detects neutral features but also provides regionally and functionally explainable findings. This allows physicians to correlate specific EEG findings with clinical presentations, offering a more comprehensive understanding of brain activity in conditions such as stress, cognitive effort, or pathological states. Moreover, this approach reduces the potential for errors that physicians may encounter when interpreting EEG data manually.
- We have generated the cortical connectome diagrams of mental performance detection and stress detection by deploying DLob sentences and transition tables. In this aspect, the cortical connectome diagrams generated illustrate transitions of dynamic brain interactions and capture dynamic shifts in brain activity. The cortical connectome diagrams showcase shifts between cortical regions, for instance, FL to TL. Each transition has a meaning according to neuroscience; for instance, FL to TL highlights a transition from cognitive or internal activity to auditory activity. To explain all transitions, we plan to present causal connectome theory (CCT) with transition triggers. However, in this research, we only demonstrated transitions for mental performance detection and stress detection. By using the interpretable results generated by DLob, we demonstrate the brain’s ability to shift focus and reveal functional connectivity between regions. Additionally, high transitions highlight robust inter-regional communication. Transition graphoelements capture shifts between regions.

Advantages:

- The recommended CubicPat-based XFE model is a highly accurate feature engineering model.
- The results of our model are obtained by deploying a 10-fold CV and LOSO CV. It demonstrates the robustness and reliability of the recommended model.
- Our model is flexible and works with both 14-channel and 32-channel EEG systems. It showcases that the CubicPat-based model is adaptable to different experimental setups and data collection tools.
- By integrating the DLob symbolic language into this feature engineering, an XFE model is presented.
- The cortical connectome diagrams for both mental performance detection and stress detection have been extracted to provide insights about neuroscience.
- This model bridges computational models with brain mechanisms. Our main idea is that we can compute feelings using the CubicPat-centric XFE model.

Limitations:

- The two used datasets have two classes. More diverse and bigger datasets can be used to detect mental performance and stress.
- Others, for instance, emotion detection and epilepsy detection, can be used to test the efficiency of the presented CubicPat-based XFE model.

Future works:

- We are planning to collect more diverse and bigger EEG signal datasets.
- The presented CubicPat-based model can be tested using more EEG signal datasets.
- By using the FNIRS device, a new generation symbolic of language and more explainable results will be presented.
- For the real-world applications in education and psychiatry, intelligent applications will be developed by using the CubicPat-based XFE model.
- The introduced CubicPat-based XFE could be applied in longitudinal studies to track mental performance or stress over time, providing insights into how brain activity patterns evolve, especially in clinical settings for the monitoring of diseases such as dementia or chronic stress.
- New generation XFE models will be presented. Based on these XFE models, a new generation of explainable deep learning models will be presented.
- By using CubicPat, like a feature extractor, a new explainable deep learning model will be presented.

## 5. Conclusions

The EEG signals related to mental performance and stress detection are classified using the presented CubicPat-based XFE model, which achieves both high classification performance and explainable results. By introducing the CubicPat feature extractor, the EEG channels are encoded by converting them into a three-dimensional feature vector, as the presented CubicPat generates the feature vector using a three-pass transition table. The most meaningful features are selected using a self-organized feature selector, the CWINCA feature selector, and these features are classified using a self-organized classifier, tkNN.

Due to the self-organized structure of the classification, high classification performance is achieved. The presented CubicPat-based XFE model achieves 99.70% accuracy with 10-fold CV and 87.79% with LOSO CV on the mental performance dataset, while the accuracies are 96.29% with 10-fold CV and 76.17% with LOSO CV for the stress detection dataset. Simultaneously, explainable results are extracted using the selected features.

With the integration of the DLob symbolic language, which provides valuable insights into brain activity, cortical connectome diagrams are generated, contributing to connectome theory. According to these results, the presented CubicPat-based model clearly demonstrates the functionality of innovative feature engineering models in the field of EEG signal classification by achieving high classification performance and explainable results on two EEG signal datasets. In the interpretable results, activations and transitions have been defined. Activation and transition in the resulting DLob strings serve as the basic concepts in the CubicPat-based XFE model and allow us to obtain a detailed and interpretable analysis of EEG signals. While activation indicates important neural interactions, transition, as shown via the cortical connectome diagram, indicates the dynamic changes between cortical regions and thus contributes to the connectome theory by showcasing transition patterns for mental performance and stress detection. Together, these concepts facilitate a comprehensive understanding of brain activity patterns and combine the computational capability of the XFE model with the complex pathophysiological mechanisms of the brain.

Additionally, AI-driven findings for neuroscience have been obtained with this model. In the future, it is planned to further develop these models to create systems capable of reading EEG signals more clearly with the CubicPat-based XFE model, enabling smart assistants in physical environments to generate EEG-explainable reports from these signals.

The key findings of this research include the introduction of the CubicPat feature extractor, and this feature extraction function (CubicPat) encodes EEG signals into three-dimensional feature vectors to provide highly informative representations. The CubicPat-based XFE model demonstrated high classification accuracies on the tested mental performance and stress detection datasets. Additionally, explainable results were achieved through the integration of the DLob symbolic language.

Improvements for future work include testing the presented CubicPat-driven XFE model on more diverse EEG signal datasets to further establish its generalizability. Future versions of the introduced CubicPat-driven model aim to increase biological interpretability by utilizing improved versions of the DLob and extending its application to other modalities, such as fNIRS. Real-time EEG report generation capabilities can also be developed to assist professionals in practical scenarios. Finally, longitudinal studies using the CubicPat-related XFE model could provide valuable insights into temporal changes, particularly in areas like mental performance tracking and stress monitoring.

## Figures and Tables

**Table 1:** The parameters of the presented CubicPat-based XFE model for this research.

Phase	Method	Parameters
Feature extraction	CubicPat	Input: Channels of the EEG signals, Qualification function: Descending, Coding: Deploying three channel values, Feature extraction method: Histogram extraction, Length of the feature vector: 32,768 for 32 channels (EEG Mental Performance Detection), 2744 for 14 channels (EEG stress detection).
Feature selection	CWINCA	Input: The extracted feature vector, Thresholds: 0.75 and 0.99, Accuracy calculator: kNN with 10-fold CV, Selection factor: Maximum accuracy, The length of the selected feature vectors: 791 for 32 channels (EEG Mental Performance Detection), 214 for 14 channels (EEG Stress Detection).
Classification	tkNN	Input: The chosen feature vector, k: from 1 to 5, Distance: City block, Chebyshev, Euclidean, Weight: Equal and Inverse, Majority voting: IMV, Number of the parameter-based outcomes: 30, Range of the IMV’s loop: from 3 to 30, The qualification criteria: Classification accuracy in descending sorting, Number of the voted outcome: 28, Selection factor: Maximum accuracy.
XAI	DLob	Input: The indices of the chosen feature vector Number of the used DLob symbol: 14, Statistical analysis: Histogram extraction, information entropy calculation and transition table computation, Graphical outcome: Cortical connectome diagram, Results generation: k-shot learning-based LLM decoder.

**Table 2:** The computed results (%).

Metric	Mental Performance		Stress	
	10-Fold CV	LOSO CV	10-Fold CV	LOSO CV
Accuracy	99.70	87.79	96.29	76.17
F1-score	99.79	91.58	96.22	75.10
Geometric mean	99.62	82	96.30	76.07

**Table 3:** Transitions of the symbols for stress detection.

	FL	FR	TL	TR	PL	PR	OL	OR
FL	**90**	23	13	4	8	8	13	8
FR	18	72	6	5	10	5	6	2
TL	9	3	30	7	9	5	7	6
TR	9	5	5	9	3	3	2	3
PL	13	5	7	6	27	2	6	3
PR	11	4	5	4	1	18	2	4
OL	7	7	7	2	8	4	25	7
OR	10	5	2	2	3	4	6	18

**Table 4:** The comparisons with SOTA models.

Research	Model	Dataset	Split Ratio	Results
Cambay et al. [7]	Feature extraction with QuadTPat, feature selection deploying CWNCA, tkNN-based classification and DLob-based interpretable results generation	Stress detection with EEG signal (14 channels)	1. 10-fold CV 2. LOSO CV	1. Acc: 92.94% 2. Acc: 73.63%
Mane and Shinde [15]	Fast Fourier transform, CNN	Stress detection with EEG signal (32 channels)	80:20	Acc: 93.20% Sen: 78.00% Spe: 81.00% F1: 76.00%
Marthinsen et al. [16]	Genetic algorithm, CNN, SVM	Stress detection with EEG signal (32 channels)	10-fold CV	Acc: 87.50% Sen: 81.25% Spe: 92.05%
Hafeez and Shakil [18]	Long- and short-term memory network, CNN	Stress detection with EEG signal (10 channels)	66.67:33.33	Acc: 90.46%
Saini et al. [19]	One-dimensional CNN	Mental tasks with EEG signal (23 channels)	10-fold CV	Acc: 99.70%
Zeng et al. [20]	Long and short-term memory network, SVM	Driver mental states detection with EEG signal	80:20	Acc: 92.68%
Wu et al. [21]	Long- and short-term memory network, CNN	Mental fatigue assessment with EEG signal (30 channels)	75:25	Acc: 99.20%
Ye et al. [22]	Ensemble CNN	Mental fatigue levels detection with EEG signal (14 channels)	90:10	Acc: 87.69%
Lee et al. [23]	Multifeature block-based CNN	Pilots’ mental states detection with EEG signal (30 channels)	2-fold CV	Acc: 75.00%
Dairi et al. [24]	CNN	Mental Tasks Recognition	80:20	AcC: 98.50
Jiang et al. [25]	Random forest-CNN	Pilots’ at-risk cognitive competency identification	75:25	Acc: 87.70%
Our model	CubicPat-based XFE	Stress detection with EEG signal (14 channels)	1. 10-fold CV 2. LOSO CV	1. Acc: 96.29% 2. Acc: 76.17%
		Mental performance detection with EEG signal (32 channels)		1. Acc: 99.70%, 2. Acc: 87.79%

**Table 5:** The CCT table.

Transition	Explanation	Medical Example
Frontal–Frontal	Indicates sustained cognitive functions.	Observed during neuropsychological tasks like the Stroop test, which evaluates executive functioning in ADHD or frontal lobe damage.
Frontal–Temporal	Reflects the integration of logical reasoning (frontal) with auditory processing and memory recall (temporal).	Seen in language tasks for Broca’s aphasia patients, where frontal regions coordinate with temporal areas for speech production.
Frontal–Parietal	It occurs during problem-solving or coordinating motor responses.	Critical in tasks requiring motor control in stroke rehabilitation or in assessing apraxia where spatial awareness is impaired.
Frontal–Occipital	This transition is critical in tasks requiring visual analysis and decision-making.	Seen during visual problem-solving tasks, such as interpreting visual puzzles in patients with traumatic brain injury (TBI).
Temporal–Frontal	This is common during emotional regulation or verbal planning.	Observed in patients with PTSD during emotional processing or in cases of auditory hallucinations in schizophrenia.
Temporal–Temporal	This transition occurs in tasks involving verbal memory or comprehension of auditory inputs.	Seen in epilepsy patients with temporal lobe seizures during memory or auditory testing.
Temporal–Parietal	It may occur when linking sounds to spatial environments.	Observed in patients with spatial neglect or auditory processing disorders during sound localization tasks.
Temporal–Occipital	This is significant in tasks requiring multimodal sensory integration (e.g., audiovisual).	Seen in cases of audiovisual integration deficits, such as in autism spectrum disorder (ASD).
Parietal–Frontal	It is critical during goal-directed behaviors.	Observed in motor planning during occupational therapy for stroke patients with hemiparesis.
Parietal–Temporal	It is seen in activities requiring spatial sound perception or object localization.	Seen in patients with vestibular disorders during balance and spatial orientation assessments.
Parietal–Parietal	This transition is active during movement planning or proprioception.	Critical in assessing proprioceptive deficits in Parkinson’s disease or peripheral neuropathy.
Parietal–Occipital	It is seen during tasks requiring visual-spatial reasoning or attention to visual stimuli.	Observed during visual-spatial reasoning tasks in patients with Balint’s syndrome or in visual attention assessments.
Occipital–Frontal	This transition is common in tasks requiring analysis of visual data for actions.	Seen in patients with visual impairments during decision-making tasks involving environmental navigation (e.g., blind individuals).
Occipital–Temporal	It occurs in scenarios requiring audiovisual integration, such as understanding lip movements in speech.	Observed in speechreading tasks for patients with hearing loss or cochlear implants.
Occipital–Parietal	It is significant in navigation or tasks requiring hand-eye coordination.	Critical in hand-eye coordination tasks for patients with optic ataxia or during rehabilitation for strokes affecting these regions.
Occipital–Occipital	It is important for analyzing complex visual stimuli or maintaining focus on visual tasks.	Seen in patients with visual agnosia during tasks requiring object recognition or shape discrimination.

## Data Availability

The authors are committed to making the data available if requested by the journal.
